# GACNNMDA: a computational model for predicting potential human microbe-drug associations based on graph attention network and CNN-based classifier

**DOI:** 10.1186/s12859-023-05158-7

**Published:** 2023-02-02

**Authors:** Qing Ma, Yaqin Tan, Lei Wang

**Affiliations:** 1 School of Software and Information Engineering, Hunan Software Vocational and Technical University, Xiangtan, 411108 China; 2 Big Data Innovation and Entrepreneurship Education Center of Hunan Province, Changsha University, Changsha, 410022 China; 3 Key Laboratory of Hunan Province for Internet of Things and Information Security, Xiangtan University, Xiangtan, 411105 China

**Keywords:** Microbe-drug associations, Graph attention network, Convolutional neural network, Computational model, Prediction model

## Abstract

As new drug targets, human microbes are proven to be closely related to human health. Effective computational methods for inferring potential microbe-drug associations can provide a useful complement to conventional experimental methods and will facilitate drug research and development. However, it is still a challenging work to predict potential interactions for new microbes or new drugs, since the number of known microbe-drug associations is very limited at present. In this manuscript, we first constructed two heterogeneous microbe-drug networks based on multiple measures of similarity of microbes and drugs, and known microbe-drug associations or known microbe-disease-drug associations, respectively. And then, we established two feature matrices for microbes and drugs through concatenating various attributes of microbes and drugs. Thereafter, after taking these two feature matrices and two heterogeneous microbe-drug networks as inputs of a two-layer graph attention network, we obtained low dimensional feature representations for microbes and drugs separately. Finally, through integrating low dimensional feature representations with two feature matrices to form the inputs of a convolutional neural network respectively, a novel computational model named GACNNMDA was designed to predict possible scores of microbe-drug pairs. Experimental results show that the predictive performance of GACNNMDA is superior to existing advanced methods. Furthermore, case studies on well-known microbes and drugs demonstrate the effectiveness of GACNNMDA as well. Source codes and supplementary materials are available at: https://github.com/tyqGitHub/TYQ/tree/master/GACNNMDA

**Supplementary Information:**

The online version contains supplementary material available at 10.1186/s12859-023-05158-7.

## Background

Researches show that Microorganisms play an integral and often unique role in human beings [1]. The microbiota and its metabolites are essential to the regulation of the host metabolism and immunity [2]. Microbes have a great impact on human health in many ways, including resistance to the invasion of opportunistic pathogens [3], promotion of the synthesis of sugar metabolism and synthesis of the necessary vitamins to boost T-cell responses [4], etc. In recent years, different aspects of the microbiome and its potential role in human health, including the early life and specific diseases, have been widely reported. For instance, Sprockett et al. explored how priority effects might influence microbial communities in the gastrointestinal tract during early childhood [5]. Ximenez et al. discussed the development of microbiota during the early times of life, from pregnancy to delivery to the early years after birth [6]. And in addition, it has been demonstrated that the intestinal microbiota plays a key role in cardiometabolic disorders, inflammatory bowel diseases, neuropsychiatric diseases and cancer separately [7–12]. Moreover, bacteria and viruses have been proven to be able to cause infectious diseases such as COVID-19 as well [13].

Simultaneously, studies show that when using drugs to treat diseases, not only the administration of drugs can affect the microbiome, but also microbial metabolism can significantly affect the clinical response of drugs [14, 15]. For example, penicillin is an important antibiotic with high efficiency and has treated pneumonia, meningitis, endocarditis, diphtheria, anthrax and so on. However, the widespread use of antibiotics has led to the development of resistance in human microbes such as staphylococcus aureus and Escherichia coli. As a result, there is an urgent need to uncover potential associations between microbes and drugs for drug development. Considering that traditional bio-experiments are quite expensive and time-consuming, it is meaningful to develop calculation models to infer possible associations between microbes and drugs, because these models can be used to guide the experimental designs of wet-lab experiments efficiently.

With the development of bioinformatical technologies, in recent years, several well-known public microbe-drug association databases such as MDAD [16], aBiofilm [17] and Drugvirus [18] have been constructed successively. Based on these databases, researchers around the world have proposed a large number of prediction methods that can be utilized to identify latent associations between microbe-drug pairs. For example, though introducing the KATZ metric to detect possible associations between microbe-drug pairs, Zhu et al. designed a prediction model named HMDAKATZ [19]. By integrating the metapath2vec scheme with a bipartite network recommendation algorithm, Long et al. proposed a computational approach called HNERMDA to infer microbe-drug associations [20]. Additionally, in 2021, Zhu et al. introduced a novel Laplacian Regularized Least Square based prediction method called LRLSMDA, which can discover latent associations between microbe-drug pairs effectively [21]. In the literature [22], through combining the graph convolutional network (GCN) with the conditional random field (CRF), Long et al. conceived a calculative model named GCNMDA to predict possible microbe-drug associations. In the literature [23], Long et al. constructed a framework of graph attention networks called EGATMDA for latent microbe–drug association prediction. Furthermore, In 2022, Deng et al. designed a multi-modal variational graph embedding model named Graph2MDA for prediction of possible microbe–drug associations [24].

Inspired by above methods, through combining the graph attention network (GAT) with a convolutional neural network (CNN)-based classifier, we proposed a novel computational model called GACNNMDA to discover potential microbe-drug associations in this manuscript. In GACNNMDA, through combining multiple measures of similarity of microbes and drugs, with known microbe-drug associations or known microbe-disease-drug associations respectively, we constructed two heterogeneous microbe-drug networks first. And then, by leveraging multiple types of microbe and drug features, we established two feature matrices for microbes and drugs simultaneously. Thereafter, after inputting these two feature matrices and two heterogeneous microbe-drug networks into a two-layer graph attention network (GAT), we obtained low dimensional feature representations for microbes and drugs respectively. Finally, we designed a convolutional neural network (CNN)-based classifier to predict possible scores of microbe-drug pairs, by integrating low dimensional feature representations and two feature matrices to form the inputs. Moreover, in order to verify the predictive performance of GACNNMDA, we performed intensive comparison experiments and case studies. Experimental results demonstrated that GACNNMDA outperformed existing representative competitive methods, and can achieve satisfactory performances in latent microbe-drug association prediction.

## Data sources

Firstly, we will download known microbe-drug associations from the database MDAD (http://www.chengroup.cumt.edu.cn/MDAD/), which includes 2470 clinically or experimentally verified microbe-drug associations between 1373 drugs and 173 microbes.

Secondly, we will download known associations among microbes, drugs and diseases from the dataset collected by Wang et al. [25], which consists of 70,315 known drug-disease associations and 15,633 known microbe-disease associations. After removing those associations associated with diseases that have no known association with any drug or microbe included in MDAD, we obtained 1121 different drug-disease associations between 233 drugs and 109 diseases, and 402 different microbe-disease associations between 73 microbes and 109 diseases respectively.

Finally, from the dataset constructed by Deng et al. [24], we collected 5586 known drug-drug interactions covering 1228 drugs in MDAD, and 138 microbe-microbe interactions covering 123 microbes in MDAD, separately. Details of these aforementioned data were shown in the following Table 1.

**Table 1:** Details of our downloaded data

Type	Microbes	Drugs	Diseases	Associations
Microbe-drug associations	173	1373	–	2470
Microbe-disease associations	73	–	109	402
Drug-disease associations	–	233	109	1121
Drug–drug interactions	–	1228	–	5586
Microbe–microbe interactions	123	–	–	138

For convenience, all these newly downloaded datasets of diseases, drugs, microbes, druf-disease associations, drug-drug interactions, microbe-drug associations, microbe-disease associations and microbe-microbe interactions will be kept in Additional files 1–8 separately.

## Methods

**Fig. 1:**
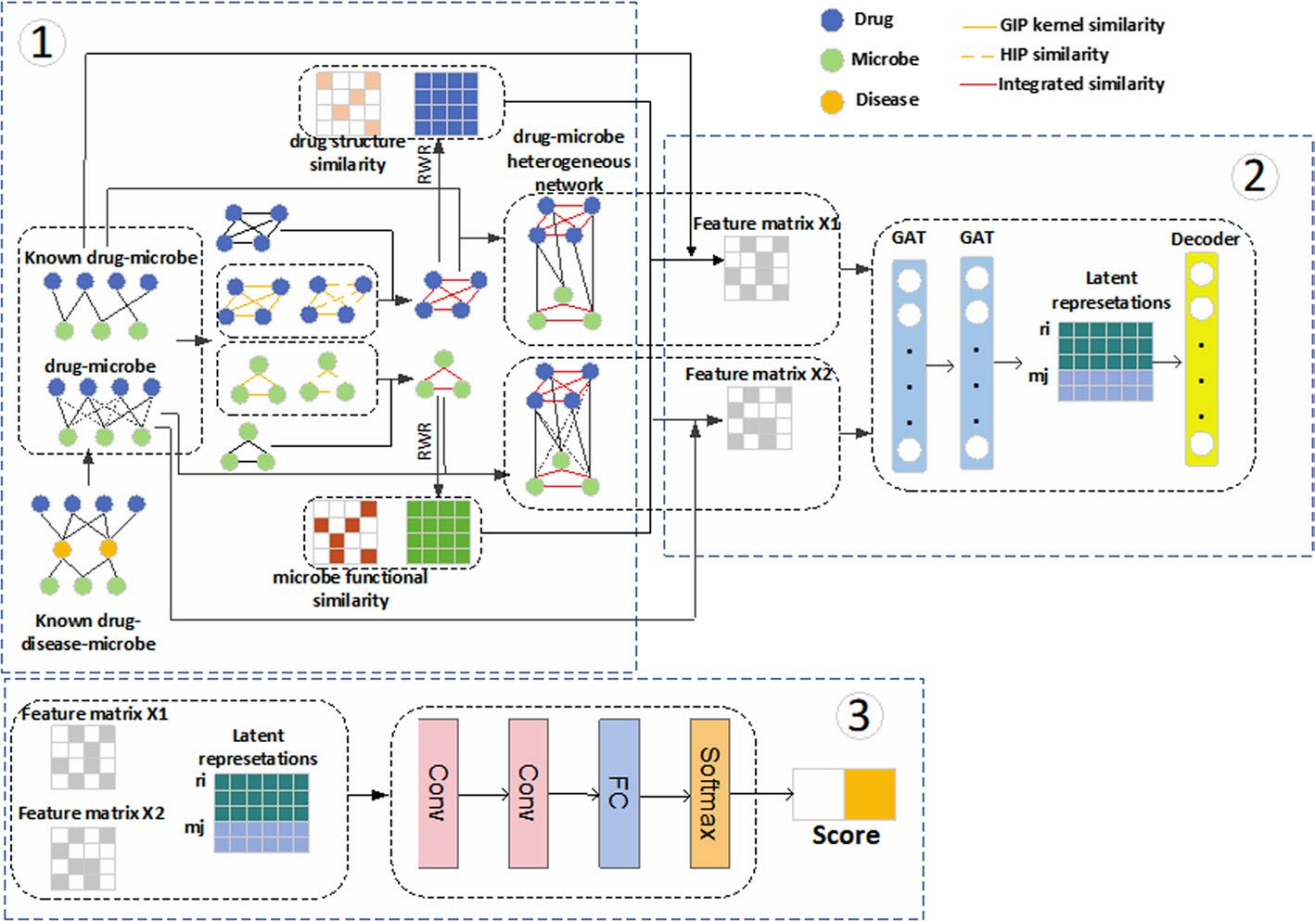
Flowchart of the GACNNMDA

As shown in Fig. 1, GACNNMDA mainly consists of three parts:

Part 1: In this part, through adopting multiple measures of similarity, two heterogenous networks *HN*_1_ and *HN*_2_ will be constructed based on downloaded known microbe-drug associations, drug-drug interactions and microbe-microbe interactions.

Part 2: In this part, two feature matrices will be obtained for microbes and drugs by leveraging various attributes of microbes and drugs first, and then, through taking these two feature matrices and two heterogeneous networks as inputs, a two-layer graph attention network will be further designed to learn low dimensional feature representations for microbes and drugs.

Part 3: In this part, a CNN-based classifier will be introduced to calculate possible scores of drug-microbe associations, in which, those newly learned low dimensional feature representations will be integrated with those two feature matrices to form its inputs.

### Construction of two heterogeneous networks

For convenience, let *n*_*r*_ and *n*_*m*_ represent the numbers of those newly downloaded drugs and microbes separately. Firstly, based on those newly downloaded known microbe-drug associations, we can obtain a microbe-drug adjacency matrix $A^{1} \in R^{{n_{r} *n_{m} }}$ as follows: for any given drug *r*_*i*_ and microbe *m*_*j*_, if there is a known association between them, then there is $A^{1} \left( {i,j} \right) = 1$, otherwise there is $A^{1} \left( {i,j} \right) = 0$.

Secondly, based on those newly downloaded known microbe-drug, microbe-disease and drug-disease associations, we can obtain another microbe-drug adjacency matrix $A^{2} \in R^{{n_{r} *n_{m} }}$ as follows: for any given drug *r*_*i*_, microbe *m*_*j*_ and disease *d*_*k*_, if there is a known association between *r*_*i*_ and *d*_*k*_, and a known association between *m*_*j*_ and *d*_*k*_, simultaneously, then there is $A^{2} \left( {i,j} \right) = 1$, otherwise there is $A^{2} \left( {i,j} \right) = A^{1} \left( {i,j} \right)$.

Finally, based on above matrices ${A}^{1}$ and ${A}^{2}$, we can construct two heterogeneous networks *HN*_1_ and *HN*_2_ respectively according to the methods proposed in the following “Calculation of the Gaussian interaction profile (GIP) kernel similarity for microbes and drugs” to “Calculation of the Gaussian interaction profile (GIP) kernel similarity for microbes and drugs” sections.

Let $A^{v} \left( {r_{i} } \right)$ and $A^{v} \left( {m_{j} } \right)$ denote the $i$-th row and the $j$-th column of $A^{v}$ (*v* = 1,2) respectively, and $\left\| \bullet \right\|$ represent the Frobenius norm, then for any two given drugs $r_{i}$ and $r_{j}$, we can calculate the GIP kernel similarity between them as follows:

1 $$S_{rg}^{v} \left( {r_{i} ,r_{j} } \right) = exp\left( { - \gamma 1\left| {\left| {A^{v} \left( {r_{i} } \right) - A^{v} \left( {r_{j} } \right)} \right|} \right|^{2} } \right)$$

2 $$\gamma 1 = 1/\left( {\frac{1}{{n_{r} }}\mathop \sum \limits_{i = 1}^{{n_{r} }} \left| {\left| {A^{v} \left( {r_{i} } \right)} \right|} \right|^{2} } \right)$$

According to above equations, it is easy to see that we can obtain a new GIP kernel similarity matrix $S_{rg}^{v} \in R^{{n_{r} {*}n_{r} }}$.

Similarly, for any two given microbes $m_{i}$ and $m_{j}$, we can calculate the GIP kernel similarity between them as follows:

3 $$S_{mg}^{v} \left( {m_{i} ,m_{j} } \right) = exp\left( { - \gamma 2\left| {\left| {A^{v} \left( {m_{i} } \right) - A^{v} \left( {m_{j} } \right)} \right|} \right|^{2} } \right)$$

4 $$\gamma 2 = 1/\left( {\frac{1}{{n_{m} }}\mathop \sum \limits_{i = 1}^{{n_{m} }} \left| {\left| {A^{v} \left( {m_{i} } \right)} \right|} \right|^{2} } \right)$$

According to above equations, it is obvious that we can obtain a new GIP kernel similarity matrix $S_{mg}^{v} \in R^{{n_{m} *n_{m} }}$.

#### Calculation of the Hamming interaction profile (HIP) similarity for microbes and drugs

Based on the assumption that two nodes will have lower similarity when their interaction profiles are more different. Let |•| denote the number of elements in the profile, then for any two given drugs *r*_*i*_ and *r*_*j*_, we can calculate the HIP similarity between them as follows:

5 $$S_{rh}^{v} \left( {r_{i} ,r_{j} } \right) = 1 - \frac{{\left| {A^{v} \left( {r_{i} } \right)! = A^{v} \left( {r_{j} } \right)} \right|}}{{\left| {A^{v} \left( {r_{i} } \right)} \right|}}$$

where $\left| {A\left( {r_{i} } \right)! = A\left( {r_{j} } \right)} \right|$ denotes the number of different elements between the profiles *A* $\left( {r_{i} } \right)$ and $A\left( {r_{j} } \right)$.

Similarly, for any two given microbes *m*_*i*_ and *m*_*j*_, we can calculate the HIP similarity between them as follows:

6 $$S_{mh}^{v} \left( {m_{i} ,m_{j} } \right) = 1 - \frac{{\left| {A^{v} \left( {m_{i} } \right)! = A^{v} \left( {m_{j} } \right)} \right|}}{{\left| {A^{v} \left( {m_{i} } \right)} \right|}}$$

where $\left| {A\left( {m_{i} } \right)! = A\left( {m_{j} } \right)} \right|$ denotes the number of different elements between the profiles *A* $\left( {m_{i} } \right)$ and $A\left( {m_{j} } \right)$.

According to above equations, it is obvious that we can obtain two new HIP similarity matrices $S_{rh}^{v} \in R^{{n_{r} *n_{r} }}$ and $S_{mh}^{v} \in R^{{n_{m} *n_{m} }}$ separately.

#### Integrated similarity

Based on $S_{rg}^{v}$, $S_{rh}^{v}$ and newly downloaded known drug-drug interactions, for any two given drugs *r*_*i*_ and *r*_*j*_, we can calculate an integrated similarity between them as follows:

7 $$S_{r}^{v} \left( {r_{i} ,r_{j} } \right) = \left\{ {\begin{array}{*{20}l} {1: } \hfill & {if\;there\;is\;a\;known\;association\;between\;r_{i} \;and\;r_{j} } \hfill \\ {\frac{{S_{rg}^{v} \left( {r_{i} ,r_{j} } \right) + S_{rh}^{v} \left( {r_{i} ,r_{j} } \right)}}{2}:} \hfill & {otherwise} \hfill \\ \end{array} } \right.$$

In the same way, based on $S_{mg}^{v}$, $S_{mh}^{v}$ and newly downloaded known microbe-microbe interactions, for any two given microbes *m*_*i*_ and *m*_*j*_, we can calculate an integrated similarity between them as follows:

8 $$S_{m}^{v} \left( {m_{i} ,m_{j} } \right) = \left\{ {\begin{array}{*{20}l} {1:} \hfill & {if\;there\;is\;a\;known\;association\;between\;m_{i} \;and\;m_{j} } \hfill \\ {\frac{{S_{mg}^{v} \left( {m_{i} ,m_{j} } \right) + S_{mh}^{v} \left( {m_{i} ,m_{j} } \right)}}{2}:} \hfill & {otherwise} \hfill \\ \end{array} } \right.$$

Hence, based on above newly obtained matrices, we can obtain two new matrices $H^{1} \in R^{{\left( {n_{r} + n_{m} } \right)*\left( {n_{r} + n_{m} } \right)}}$ and $H^{2} \in R^{{\left( {n_{r} + n_{m} } \right)*\left( {n_{r} + n_{m} } \right)}}$ as follows:

9 $$H^{1} = \left[ {\begin{array}{*{20}c} {S_{r}^{1} } & {A^{1} } \\ {(A^{1} )^{T} } & {S_{m}^{1} } \\ \end{array} } \right]$$

10 $$H^{2} = \left[ {\begin{array}{*{20}c} {S_{r}^{2} } & {A^{2} } \\ {(A^{2} )^{T} } & {S_{m}^{2} } \\ \end{array} } \right]$$

Obviously, according to above two matrices $H^{1}$ and $H^{2}$, we can easily construct two heterogeneous networks *HN*_1_ and *HN*_2_ respectively.

### Low dimensional feature representations learning for microbes and drugs based on the graph attention network

#### Construction of two feature matrices

In this section, for any two given drugs *r*_*i*_ and *r*_*j*_, we would first adopt SIMCOMP2 [26] to calculate the structural similarity between them, as a result, we can obtain a new drug structural similarity matrix $S_{rc}$. And at the same time, for any two given microbes *m*_*i*_ and *m*_*j*_, we would adopt the method proposed by Kamneva et al. [27] to calculate the functional similarity between them, as a result, we can obtain a new microbe functional similarity matrix $S_{mf}$ as well.

Moreover, we would further implement a random walk with restart (RWR) on $S_{r}^{v}$ and $S_{m}^{v}$ to obtain the topological attributes $S_{rr}^{v} , S_{mm}^{v}$ of drugs and microbes separately, where the RWR was defined as follows:

11 $$p_{i}^{l + 1} = 0.1*Mp_{i}^{l} + 0.9*\varepsilon_{i}$$

12 $$\varepsilon_{ij} = \left\{ {\begin{array}{*{20}c} {1 if i = j} \\ {0 otherwise} \\ \end{array} } \right.$$

Here, $p_{i}^{l}$ denotes the probabilities that node $i$ reaches other nodes at the time slot $l$. *M* is the transition probability matrix and $\varepsilon_{i} \varepsilon R^{1*n}$ represents the initial probability vector of node $i$.

Different from the usual weighted addition of various attribute vectors of nodes to form the feature matrix, we spliced various attributes together to retain more original features. The feature matrices $X^{v} \in R^{{\left( {n_{r} + n_{m} } \right)*k_{1} }}$ for two heterogeneous networks were defined as follows:

13 $$F_{r}^{v} = \left[ {S_{rc} ;A^{v} ;S_{rr}^{v} ;A^{v} } \right]$$

14 $$F_{m}^{v} = \left[ {\left( {A^{v} } \right)^{T} ;S_{mf} ;\left( {A^{v} } \right)^{T} ;S_{mm}^{v} } \right]$$

15 $$X^{v} = \left[ {\begin{array}{*{20}c} {F_{r}^{v} } \\ {F_{m}^{v} } \\ \end{array} } \right]$$

where $k_{1}$ denotes the dimension of the feature matrices $X^{v}$.

#### The structure of the graph attention network

*Encoder*: Firstly, for any given node $i$ in $H^{v} \left( {v = 1,2} \right)$, the coefficient of similarity between it and its neighbors would be calculated as follows:

16 $$e_{ij} = LeakyRelu\left( {a\left[ {W^{v} X^{v} \left( i \right);W^{v} X^{v} \left( j \right)} \right]} \right), j \in {\Phi }_{i}^{v}$$

17 $$LeakyRelu\left( x \right) = \left\{ {\begin{array}{*{20}l} x \hfill & {x > 0} \hfill \\ {\mu x} \hfill & {otherwise} \hfill \\ \end{array} } \right.$$

Here, $X^{v} \left( i \right)$ denotes the *i*th row of $X^{v}$ and $a$ represents a feature mapping operation. $W^{v}$ is a trainable weight matrix parameter and $\Phi_{i}^{v}$ is the set of neighbor nodes of node $i$ in $H^{v}$, $\mu$ is the hypermeter.

Subsequently, the attention score $\lambda_{ij}$ between node $i$ and node $j$ would be calculated based on $e_{ij}$ according to the following formula:

18 $$\lambda_{ij} = \frac{{exp\left( {e_{ij} } \right)}}{{\mathop \sum \nolimits_{{k \in {\Phi v}_{i} }} exp\left( {e_{ik} } \right)}}$$

Finally, the features would be weighted and summed according to the calculated attention score to obtain the new feature representation of node $i$ as follows:

19 $$X^{v} \left( i \right)^{^{\prime}} = Relu\left( {\mathop \sum \limits_{{j \in {\Phi v}_{i} }} \lambda_{ij} W^{v} X^{v} \left( j \right)} \right)$$

20 $$Relu\left( x \right) = \left\{ {\begin{array}{*{20}l} x \hfill & {x > 0} \hfill \\ 0 \hfill & {otherwise} \hfill \\ \end{array} } \right.$$

After obtaining new feature representations of all nodes in $H^{v}$, it is easy to see that we can construct a feature representation matrix $$Y^{v} = \left[ {\begin{array}{*{20}c} {R_{r}^{v} } \\ {R_{m}^{v} } \\ \end{array} } \right] \in R^{{\left( {n_{r} + n_{m} } \right)*k_{2} }}$$.

Where $k_{2}$ denotes the dimension of the feature representation matrix $Y^{v}$.

*Decoder*: The decoder runs an inner product based on newly learned feature representation matrix ${Y}^{v}$ as follows:

21 $$Y^{v\prime } = Sigmoid\left( {Y^{v} \cdot \left( {Y^{v} } \right)^{T} } \right)$$

22 $$Sigmoid\left( x \right) = \frac{1}{{1 + e^{ - x} }}$$

#### Optimization

Considering the reconstructed matrix should be as similar as possible to the original matrix, we adopted the MSE loss function to compute the mean of the sum of squares of the differences between $Y^{v\prime }$ and $H^{v}$ as follows:

23 $$Loss = \frac{1}{{n_{r} + n_{m} }}\mathop \sum \limits_{i = 1}^{{n_{r} + n_{m} }} \left| {\left| {Y^{v\prime } \left( i \right) - H^{v} \left( i \right)} \right|} \right|^{2}$$

where $Y^{v\prime } \left( i \right)$ and $H^{v} \left( i \right)$ denote the $i$-th row of $Y^{v\prime }$ and $H^{v}$ respectively. During training, we used the Adam optimizer to optimize the loss function.

### Construction of the CNN-based classifier

In this section, we treated the microbe-drug association prediction as a binary classification problem and designed a classifier based on the convolutional neural network to calculate possible scores of potential drug-microbe associations. For the input of the classifier, we first constructed two new feature matrices $N_{r}^{v}$ and $N_{m}^{v}$ for drugs and microbes separately as follows:

24 $$N_{r}^{v} = \left[ {R_{r}^{v} ;F_{r}^{v} } \right]$$

25 $$N_{m}^{v} = [R_{m}^{v} ;F_{m}^{v} ]$$

And then, let $k_{3}$ denote the dimension of the new feature matrix, then for any given drug $r_{i}$ and microbe $m_{j}$, the feature matrix $$F^{v} \left( {i,j} \right) = \left[ {\begin{array}{*{20}c} {N_{r}^{v} \left( i \right)} \\ {N_{m}^{v} \left( j \right)} \\ \end{array} } \right] \in R^{{2*k_{3} }}$$ would be fed into the classifier to calculate the score between $i$ and $j$. Here,$N_{r}^{v} \left( i \right)$ and $N_{m}^{v} \left( j \right)$ denote the $i$-th and the $j$-th row of $N_{r}^{v}$ and $N_{m}^{v}$, respectively.

In the convolutional layer, we adopted zero padding to enlarge the edges and set the size of the convolution kernel to 3 × 3. The convolutional operation in the $i$-th layer were defined as follows:

26 $$F_{i} = Relu\left( {F_{i - 1} \otimes G_{i} + b_{i} } \right)$$

where $\otimes$ represents the operation of convolution, $G_{i}$ is the weight matrix, and $b_{i}$ is the offset vector. It is worth mentioning that we added the BatchNorm2d [28] to normalize data to enhance performance stability before $Relu$.

After inputs having gone through two convolution layers, it would be flattened into a vector. And then, a full-connected layer and a softmax layer would be used to obtain scores of two associative categories, based on which, we would adopt scores of the second category as predicted scores of potential microbe-drug associations in GACNNMDA. Obviously, based on $H^{1}$ and $H^{2}$, we can obtain two score matrices $Score^{1}$ and $Score^{2}$ respectively. Hence, a final score matrix $Score \in R^{{n_{r} *n_{m} }}$ can be calculated as follows:

27 $$Score\left( {i,j} \right) = \frac{{Score^{1} \left( {i,j} \right) + Score^{2} \left( {i,j} \right)}}{2}$$

Moreover, in the classifier, we utilized the cross-entropy as loss function and Adam optimizer to minimize the loss function. Here, the loss function $L^{v}$ (*v* = 1, 2) was defined as follows:

28 $$L^{v} = - \frac{1}{{n_{r} *n_{m} }}\sum a_{ij}^{v} logs_{ij}^{v} + \left( {1 - a_{ij}^{v} } \right)log\left( {1 - s_{ij}^{v} } \right)$$

where $a_{ij}^{v}$ and $s_{ij}^{v}$ represent the $ij$-th entry of $A^{v}$ and $Score^{v}$ respectively.

## Results

### Comparison with state-of-the-art methods

Considering that there are few computational methods and codes available for microbial-drug association prediction, we compared GACNNMDA with four existing microbe-drug association prediction methods such as HMDAKATZ [19], GCNMDA [22], EGATMDA [23] and Graph2MDA [24], and two methods for link prediction problems in the bioinformatics field such as LAGCN [29] and NTSHMDA [30]. Among them, LAGCN [29] is a graph convolutional network with attention mechanism based method designed to infer unknown drug-disease associations. NTSHMDA [30] is a model based on random walk with restart for predicting microbe-disease associations.

**Fig. 2:**
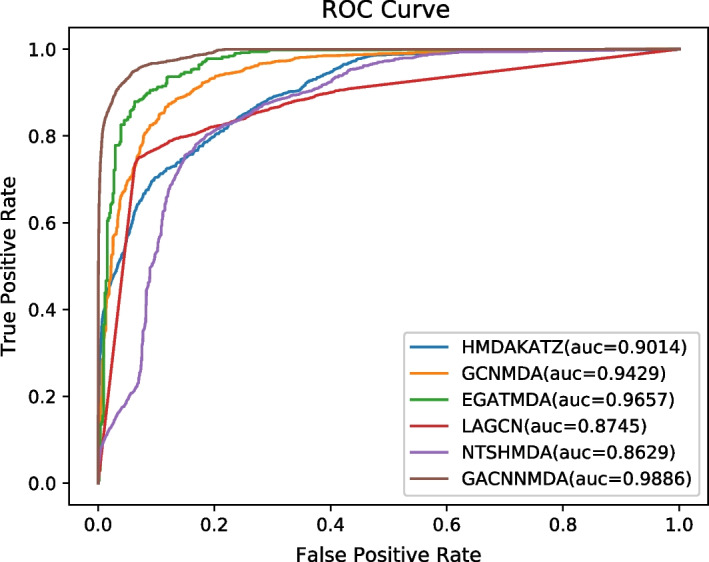
The ROC curves of six competitive methods

**Fig. 3:**
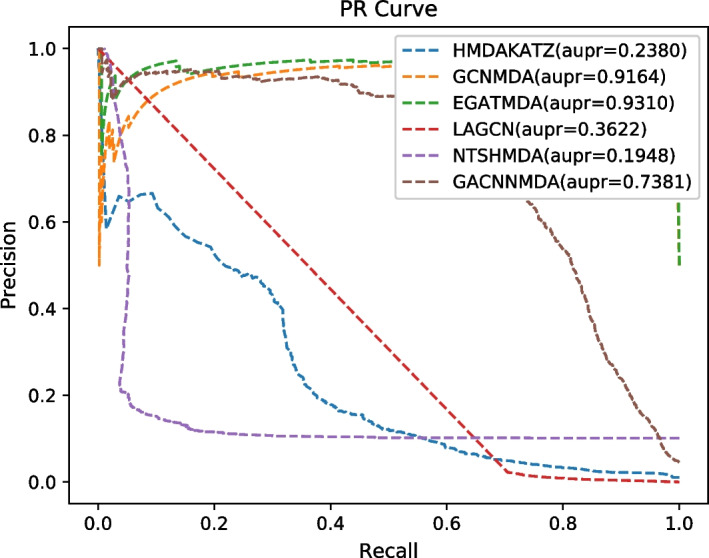
The PR curves of six competitive methods

During experiments, we settled with original parameters for all these competitive methods and ran them on the well-known public database MDAD for a fair comparison. In addition, we adopted the framework of fivefold cross validation (CV) to evaluate these methods, in which, 20% of known associations and 20% of unknown associations would be randomly selected as the testing set, and the remaining 80% of known associations and unknown associations as the training set [31]. And then, we selected the AUC, AUPR, Accuracy and F1-Score as the metrics of performance evaluation. Experimental results were shown in Table 2. Due to the incomplete code proposed by Deng et al. [24], we directly referenced the results in Graph2MDA. As a result, the ROC and PR curves were drawn in Figs. 2 and 3 separately, in which, those evaluation metrics are calculated as follows:

29 $$TPR = \frac{TP}{{TP + FN}}$$

30 $$FPR = \frac{FP}{{TN + FP}}$$

31 $$Precision = \frac{TP}{{TN + FP}}$$

32 $$Recall = \frac{TP}{{TP + FN}}$$

33 $$Accuracy = \frac{TP + TN}{{TP + TN + FP + FN}}$$

34 $$F1 - score = 2*\frac{Precision*Recall}{{Precision + Recall}}$$

**Table 2:** The AUCs, AUPRs, accuracy and F1-scores achieved by compared methods based on MDAD under fivefold CV

Methods	AUC	AUPR	Accuracy	F1-score
HMDAKATZ	0.8712 ± 0.0010	0.2327 ± 0.0068	0.9774	0.3546
GCNMDA	0.9427 ± 0.0002	0.9133 ± 0.0031	0.9905	0.6672
EGATMDA	0.9585 ± 0.0053	0.9268 ± 0.0142	0.9081	0.6871
Graph2MDA	0.9567 ± 0.0039	**0.9380 ± 0.0098**	0.9934	0.7091
LAGCN	0.8533 ± 0.0070	0.3571 ± 0.0051	0.9413	0.0423
NTSHMDA	0.8483 ± 0.0020	0.1892 ± 0.0056	0.9896	0.1838
GACNNMDA	**0.9777 ± 0.0109**	0.7015 ± 0.0366	**0.9945**	**0.7091**

Bold values indicate the best results achieved by all these competitive methods

Here, TP and TN represent the numbers of positive and negative samples predicted correctly, respectively. FN and FP denote the numbers of positive and negative samples that are incorrectly identified, separately.

As shown in Table 2, it is obvious that GACNNMDA can achieve the highest AUC value of 0.9777 ± 0.0109, which is 2.57% higher than the second highest AUC value of 0.9585 ± 0.0053 obtained by EGATMDA. For evaluation metrics of accuracy and f1-score, GACNNMDA can also achieve the highest values of 0.9945 and 0.7091 respectively. Although in terms of AUPR value, GACNNMDA can only outperform half of all these competitive methods, we can say that GACNNMDA is an effective tool for potential microbe-drug association prediction.

### Hyperparameter sensitivity analysis

Considering that there are several hyperparameters in GACNNMDA, including the learning rate of GAT, the dropout of GAT and the learning rate of CNN, therefore, in this section, we would perform a fivefold CV on the MDAD dataset for 10 times and observe the average AUC value to tune the values of these parameters.

**Fig. 4:**
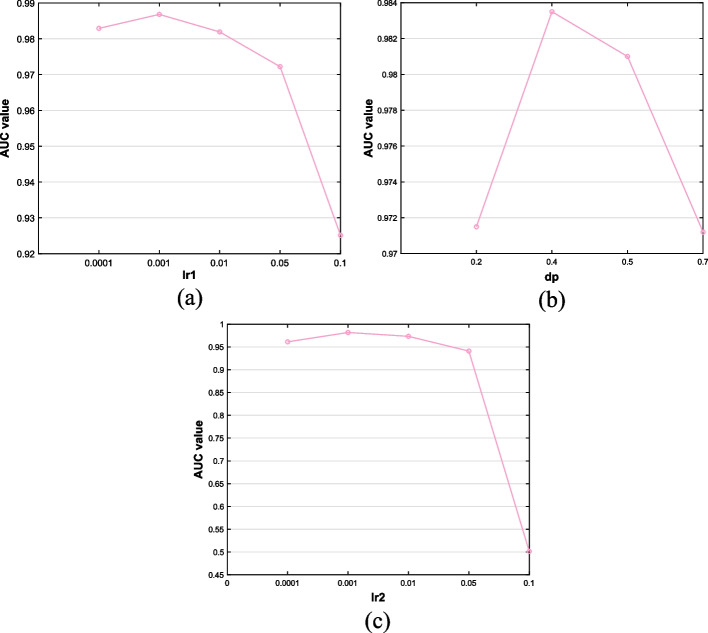
Analysis of the impact of hyperparameters on performance of GACNNMDA. The subfigures from (**a**) to (**c**) show the AUC values of related values of the learning rate of GAT, the dropout of GAT and the learning rate of CNN, respectively

For convenience, let *lr*1, *dp* and *lr*2 denote the learning rate of GAT, the dropout of GAT and the learning rate of CNN respectively. During the tuning process, we first tested the values of *lr*1 in the range of {0.0001, 0.001, 0.01, 0.05, 0.1} and illustrated experimental results in Fig. 4a. As shown in Fig. 4a, GACNNMDA achieved the best performance when *lr*1 was set to 0.001. And then, we limited the values of *dp* in the range of {0.2, 0.4, 0.5, 0.7} and illustrated experimental results in Fig. 4b. From observing Fig. 4b, it is easy to see that the most suitable value of *dp* is 0.4. Finally, we restricted the values of *lr*2 in {0.0001, 0.001, 0.01, 0.05, 0.1} and showed experimental results in Fig. 4c. As illustrated in Fig. 4c, when *lr*2 was set to 0.001, the performance of GACNNMDA would be the best.

### Case studies

In order to further demonstrate the prediction performance of GACNNMDA, case studies on two popular drugs and two microbes will be done in this section. And in experiments of case studies, the top 20 microbes or drugs inferred by GACNNMDA based on the database of MDAD will be selected out for investigation first, and then, we will search published PubMed literatures to verify whether these predicted candidates having been reported by existing references.

The first drug that we chose for case studies is Ciprofloxacin, which is a fluorinated quinolone antibiotic, and a large number of studies have shown that it is associated with a wide range of human microbes [32]. For instance, Paul et al. found that Amphotericin-B and 5% ciprofloxacin can effectively hindered the growth of Pseudomonas aeruginosa and Candida albicans [33]. *Staphylococcus aureus*, *Staphylococcus epidermidis*, *Bacillius subtilius*, *Escherichia coli* and Mycobacterium tuberculosis are susceptible to Ciprofloxacin [34]. The second drug that we chose for case studies is Moxifloxacin, which is a fluoroquinolone antibiotic [35], and has been proven to be associated with antibiotic-resistant bacteria (ARB) [36] and Listeria monocytogenes [37]. And as a result, we illustrated the top 20 predicted ciprofloxacin-associated and moxifloxacin-associated microbes in Tables 3 and 4 respectively. From observing Tables 3 and 4, it is easy to see that there are 18 and 17 out of top 20 predicted microbes having been validated by existing literatures separately.

**Table 3:** The top 20 candidate Ciprofloxacin-associated microbes

Microbe	Evidence	Microbe	Evidence
*Staphylococcus aureus*	PMID: 32488138	*Streptococcus sanguinis*	PMID: 21507381
*Mycobacterium tuberculosis*	PMID: 30020039	*Enterococcus faecalis*	PMID: 27790716
*Escherichia coli*	PMID: 26607324	*Eggerthella lenta*	Unconfirmed
*Bacillus subtilis*	PMID: 33218776	*Salmonella enterica*	PMID: 6933017
*Haemophilus influenzae*	PMID: 27292570	Human herpesvirus 5	Unconfirmed
*Stenotrophomonas maltophilia*	PMID: 14982788	*Propionibacterium acnes*	PMID: 25445201
*Pseudomonas aeruginosa*	PMID: 33875431	*Klebsiella pneumoniae*	PMID: 27257956
*Morganella morganii*	PMID: 29942700	*Staphylococcus cohnii*	PMID: 19780489
*Providencia stuartii*	PMID: 1337751	*Serratia marcescens*	PMID: 2071875
*Proteus vulgaris*	PMID: 34638966	*Staphylococcus epidermis*	PMID: 10632381

The top 10 predicted microbes are included in the first column, while the top 11–20 predicted microbes are included in the third column records

**Table 4:** The top 20 candidate Moxifloxacin-associated microbes

Microbe	Evidence	Microbe	Evidence
*Bacillus subtilis*	PMID: 30036828	*Staphylococcus Aureus*	PMID: 31689174
*Haemophilus influenzae*	PMID: 11856249	Enterococcus faecium	PMID: 10629010
*Stenotrophomonas maltophilia*	PMID: 27257956	Human herpesvirus 5	Unconfirmed
*Candida albicans*	PMID: 21108571	*Proteus vulgaris*	Unconfirmed
*Mycobacterium avium*	PMID: 21353489	Bacillus cereus	PMID: 21834669
*Pseudomonas aeruginosa*	PMID: 31691651	*Streptococcus pneumoniae*	PMID: 31542319
*Campylobacter jejuni*	PMID: 16027651	Serratia marcescens	PMID: 34439014
*Staphylococcus aureus*	PMID: 31689174	*Streptococcus mutans*	PMID: 29160117
*Neisseria gonorrhoeae*	PMID: 26603424	*Klebsiella pneumoniae*	PMID: 33406110
*Escherichia coli*	PMID: 31542319	Bacteroides	PMID: 18385145

The top 10 predicted microbes are included in the first column, while the top 11–20 predicted microbes are included in the third column records

Besides, the first microbe that we chose for case studies is HIV-1 (Human Immunodeficiency Virus type 1), which is the cause of the acquired immunodeficiency syndrome (AIDS). There are many drugs associated with HIV-1. For example, Viani et al. found that long-term zalcitabine for treating HIV-1 phenotypes in children is useful [38]. Chong et al. proved that combination of delavirdine, zidovudine and didanosine can inhibit the growth of the HIV-1 [39]. The second microbe that we chose for case studies is mycobacterium tuberculosis, which is the cause of the pulmonary tuberculosis [40]. And as a result, we showed the top 20 predicted HIV-1-associated and mycobacterium tuberculosis-associated drugs in Tables 5 and 6 respectively. From observing Tables 5 and 6, it is obvious that there are 18 and 15 out of top 20 predicted drugs having been verified by existing literatures. Hence, we can draw a conclusion that GACNNMDA can achieve satisfactory prediction performance in both case studies of microbes and drugs.

**Table 5:** The top 20 candidate Human immunodeficiency virus type 1-associated drugs

Drug	Evidence	Drug	Evidence
Zalcitabine	PMID: 9498433	Cala + B20nolide A	PMID: 8930168
Abacavir	PMID: 11797183	Tenofovir	PMID: 33336698
Fosamprenavir	PMID: 19515730	Bevirimat	PMID: 19024627
Didanosine	PMID: 9107385	Dolutegravir	PMID: 31865558
Indinavir	PMID: 8970946	Peptide 1037	Unconfirmed
Delavirdine	PMID: 9107385	Vancomycin	Unconfirmed
Tipranavir	PMID: 17360759	Nevirapine	PMID: 20384494
Stavudine	PMID: 8568296	Enfuvirtide	PMID: 14523775
Atazanavir	PMID: 15585441	Lopinavir	PMID: 20836579
Zidovudine	PMID: 2012453	Trimethoprim-sulfamethoxazole	PMID: 9142796

The top 10 predicted drugs are included in the first column, while the top 11–20 predicted drugs are included in the third column records

**Table 6:** The top 20 candidate Mycobacterium tuberculosis-associated drugs

Drug	Evidence	Drug	Evidence
Ciprofloxacin	PMID: 16270765	Meropenem	PMID: 22906310
Aminosalicylic acid	PMID: 26033719	Polysorbate 80	Unconfirmed
SQ109	PMID: 22258923	Pyrogallol	PMID: 13411428
Colistin	PMID: 26183185	Pefloxacin	PMID: 1909062
Ethambutol	PMID: 27806932	Zinc oxide	PMID: 33845951
Tobramycin	PMID: 19723387	Desipramine	PMID: 7649718
Pyrazinamide	PMID: 26521205	Saquinavir	PMID: 33841429
Telithromycin	unconfirmed	Gatifloxacin	PMID: 17267339
Capreomycin	PMID: 29311078	Undecanoic acid	Unconfirmed
Trans-2-nonenal	Unconfirmed	Piperacillin-Tazobactam	Unconfirmed

The top 10 predicted drugs are included in the first column, while the top 11–20 predicted drugs are included in the third column records

## Conclusion and discussion

In this paper, we presented a novel calculation method named GACNNMDA, an integrated framework of GAT-based autoencoder and CNN-based classifier, for prediction of potential microbe-drug associations. The main contributions of our model include the following three points.

1. We introduced known microbe-disease-drug associations into the predictive model and made up for the sparsity of known microbe-drug associations to some extent.
2. For the inputs of GAT and CNN, we spliced multiple attributes of microbes and drugs together to form two feature matrices, which can retain more original features of microbes and drugs. Hence, more useful information can be learned by the GAT and the CNN.
3. Compared with existing state-of-the-art methods for predicting potential microbe-drug associations, our model can achieve better performance.

However, there is still room to improve our prediction model. In the feature, we can leverage more biological information, such as microbe sequences [24] and side-effect-based drug similarity [41]. Additionally, for those attributes of microbes and drugs used in GACNNMDA, we can make an assessment of their importance to better use each kind of attribute and further improve the performance of our model. Finally, we can design a new activation to improve the training speed of GAT and CNN such as Li et al. [42].

## Supplementary Information

**Additional file 1:** The newly downloaded dataset of diseases.

**Additional file 2:** The newly downloaded dataset of drugs.

**Additional file 3:** The newly downloaded dataset of microbes.

**Additional file 4:** The newly downloaded dataset of drug-disease associations.

**Additional file 5:** The newly downloaded dataset of drug-drug interactions.

**Additional file 6:** The newly downloaded dataset of microbe-drug associations.

**Additional file 7:** The newly downloaded dataset of microbe-disease associations.

**Additional file 8:** The newly downloaded dataset of microbe-microbe interactions.

## Data Availability

The data and code can be found online at: https://github.com/tyqGitHub/TYQ/tree/master/ GACNNMDA.
